# High-performance polyvinyl chloride gel artificial muscle actuator with graphene oxide and plasticizer

**DOI:** 10.1038/s41598-019-46147-2

**Published:** 2019-07-04

**Authors:** Taeseon Hwang, Zachary Frank, Justin Neubauer, Kwang Jin Kim

**Affiliations:** Department of Mechanical Engineering, University of Nevada, Las Vegas, 4505 Maryland Parkway, Las Vegas, Nevada 89154 United States

**Keywords:** Mechanical engineering, Gels and hydrogels

## Abstract

A transparent and electroactive plasticized polyvinyl chloride (PVC) gel was investigated to use as a soft actuator for artificial muscle applications. PVC gels were prepared with varying plasticizer (dibutyl adipate, DBA) content. The prepared PVC gels were characterized using Fourier-transform infrared spectroscopy, thermogravimetric analysis, and dynamic mechanical analysis. The DBA content in the PVC gel was shown to have an inverse relationship with both the storage and loss modulus. The electromechanical performance of PVC gels was demonstrated for both single-layer and stacked multi-layer actuators. When voltage was applied to a single-layer actuator and then increased, the maximum displacement of PVC gels (for PVC/DBA ratios of 1:4, 1:6, and 1:8) was increased from 105.19, 123.67, and 135.55 µm (at 0.5 kV) to 140.93, 157.13, and 172.94 µm (at 1.0 kV) to 145.03, 191.34, and 212.84 µm (at 1.5 kV), respectively. The effects of graphene oxide (GO) addition in the PVC gel were also investigated. The inclusion of GO (0.1 wt.%) provided an approximate 20% enhancement of displacement and 41% increase in force production, and a 36% increase in power output for the PVC/GO gel over traditional plasticizer only PVC gel. The proposed PVC/GO gel actuator may have promising applications in artificial muscle, small mechanical devices, optics, and various opto-electro-mechanical devices due to its low-profile, transparency, and electrical response characteristics.

## Introduction

Polymer based artificial muscles have seen great interest from the research community because they demonstrate large strains, have a high response rates, and have a large power output in response to changes of external factors like temperature, pH, light, and electric field^1–5^. Electroactive polymers (EAPs) are polymers which show large strains in response to electrical stimuli. Ionic polymer-metal composites are a type of actuator that exhibit relatively large deformation with low input voltages, typically around 1–3 V, however they require hydration to function properly which poses a challenge in many applications^6^. Dielectric elastomer actuators are another type of EAP actuator where great focus has been applied and a variety of applications have been developed including fluid pumps, variable focus lenses, and conformal skins^7,8^. However, this type of actuator requires high voltages (often >1 kV) because high electric fields (~100 V/µm) are necessary for actuation^9^. Microhydraulic artificial muscles have also been heavily studied and come in a variety of forms, but they are often used in applications where larger actuators are necessary (for McKibben type hydraulic actuators), or do not have as high of a force output as other artificial muscles as is the case with microhydraulic stepping actuators^10^. Recent progress has also been made into the use of liquid dielectrics in hydraulically amplified self-healing electrostatic actuators which allow for very large displacements to be obtained with a high frequency, these are very promising for some larger applications, but the voltages used can often be on the range of 10 s of kV, which is too high for many applications^11^.

Polymer gels have garnered great interest in applications for actuators, artificial muscles, and sensors because their volume and shape can be manipulated through multiple variables such as temperature, solvent, light, pH, and electric field^12^. Most research on the electrical actuation of polymer gels have focused on polyelectrolyte gels. These gels actuate due to their ionic species content which causes deformation under an electric field via ionic migration^13,14^. However, water, salt solutions, or other ionic liquids are typically required for actuation of polyelectrolyte gels, which is a problematic constraint as it limits the applications of gels due to the occurrence of electrolysis which occurs at higher voltages and currents. Poly(vinyl alcohol)-dimethyl sulfoxide gel has also been studied as it is electrically inactive due to its lack of ionic groups. It can be deformed in air, but it has poor strength and problems with leakage^15^.

Polyvinyl chloride (PVC) gel actuators or artificial muscles have shown a significant potential for efficient applications as PVC is easily available, low-cost, and electrically inactive. Plasticized PVC gels have also shown excellent flexibility, ability to conform to irregular surfaces and have a high surface tension^16^. Additionally, PVC gels have many advantages as actuators such as their ability to move in air, large strains, high output stresses, high response rates, and stability under thermal influence^17^. It has been theorized that deformation in PVC gels is caused by electric charge injection at the cathode, plasticizer migration, and the Maxwell force caused by the electric field between two parallel electrodes^18^. PVC gel actuators exhibit large amounts of deformation and a wide range of motions that can be achieved by creating appropriate boundary conditions for the actuators, allowing the creeping motion to be utilized for different mechanisms (bending, contracting, etc.)^19^.

Graphene oxide (GO) contains carbonyl and carboxyl groups at the sheet edges and polar groups (hydroxyl and epoxide functional groups) on its basal planes; thus, GO easily can be dispersed into a polymer matrix because of the interactions between its functional groups and the polymer chains^20,21^. With these characteristics, GO has been often utilized in an electrophoretic deposition (EPD) process. EPD is a colloidal process where the suspended particles are impelled from the suspension medium to the substrate by an electric field^22^. Electrophoresis happens when the electric field is applied to the GO suspension, the charged GO move toward the oppositely charged electrode driven by the electric force^23^.

Here, we have investigated PVC gel properties and the electromechanical performance of PVC gel actuators for use as artificial muscles with varying plasticizer content (PVC to plasticizer ratio, 1:4, 1:6 and 1:8). In addition, the study was conducted to maximize the performance of PVC gels by adding GO to plasticized PVC gels using electrophoretic mobility of the GO. PVC gels with GO were also prepared to test for possible changes in electromechanical performance (0.1 and 1.0 wt.% of GO to PVC). PVC and PVC/GO gels have been prepared via a solution casting method, detailed conditions are summarized in Table 1. The thickness of prepared PVC and PVC/GO gels have been measured at approximately 1 mm. We have investigated the thermal and mechanical properties, as well as chemical structure of prepared gels and the electromechanical performance of single and multi-stacked gel actuators using in-house electronics.

**Table 1:** The summarized conditions to prepare plasticized PVC and PVC/GO gels.

Samples	PVC (g)	DBA (g)	GO (mg)	THF (ml)	Remark
PVC	1	−	−	20	Transparent
P4	1	4	−	20	Transparent
P6	1	6	−	20	Transparent
P8	1	8	−	20	Transparent
P8G01	1	8	1	20	Transparent brown
P8G1	1	8	10	20	Black

## Results and Discussion

**Figure 1:**
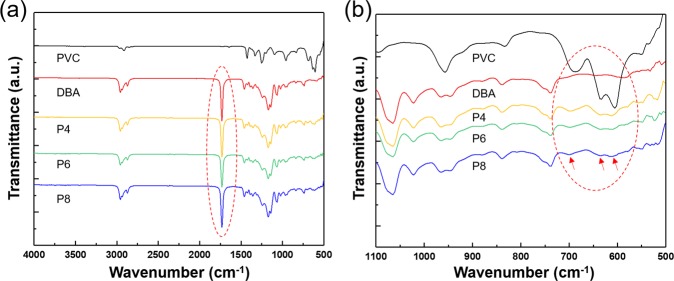
The FT-IR spectra of the PVC, DBA, and PVC gels: (**a**) Wide range spectra from 4,000 to 500 cm^−1^. (**b**) Reduced range spectra from 1,600 to 500 cm^−1^.

Figure 1a,b show the Fourier transform infrared spectroscopy (FT-IR) spectra for pristine PVC, dibutyl adipate (DBA), and PVC gels with different PVC/DBA ratios (i.e., 1:4, 1:6 and 1:8). As shown in Fig. 1a, the characteristic peaks of the PVC molecules can be seen; including peaks for C-H bands (1,427 and 1,257 cm^−1^), C-C vibrations (968 cm^−1^), and C-Cl bands (698, 636 and 605 cm^−1^)^24^. Also, the spectra show the peak at 1,732 cm^−1^ which corresponds with the carbonyl group (C = O) bands in the DBA and PVC gels. The carbonyl group is one of the major functional groups that can react to polarize or plasticize the polymer^25^. Figure 1b shows the vibration stretching of C–Cl bands of PVC at 698, 636 and 605 cm^−1^, which also appear in the prepared PVC gels (P4, P6, and P8)^26^. From these FT-IR results, both DBA and PVC are expected to be strongly polarizable due to C = O and C–Cl bands in their structures, resulting PVC gels are also expected to exhibit polarizability. The C = O bands affect solvent movement between the cathode and the anode under an applied field and the dipole moment of PVC (C–Cl) might cause molecules to be arranged under the influence of polarizable solvents [in the case of this study tetrahydrofuran (THF) was used]^27^.

**Figure 2:**
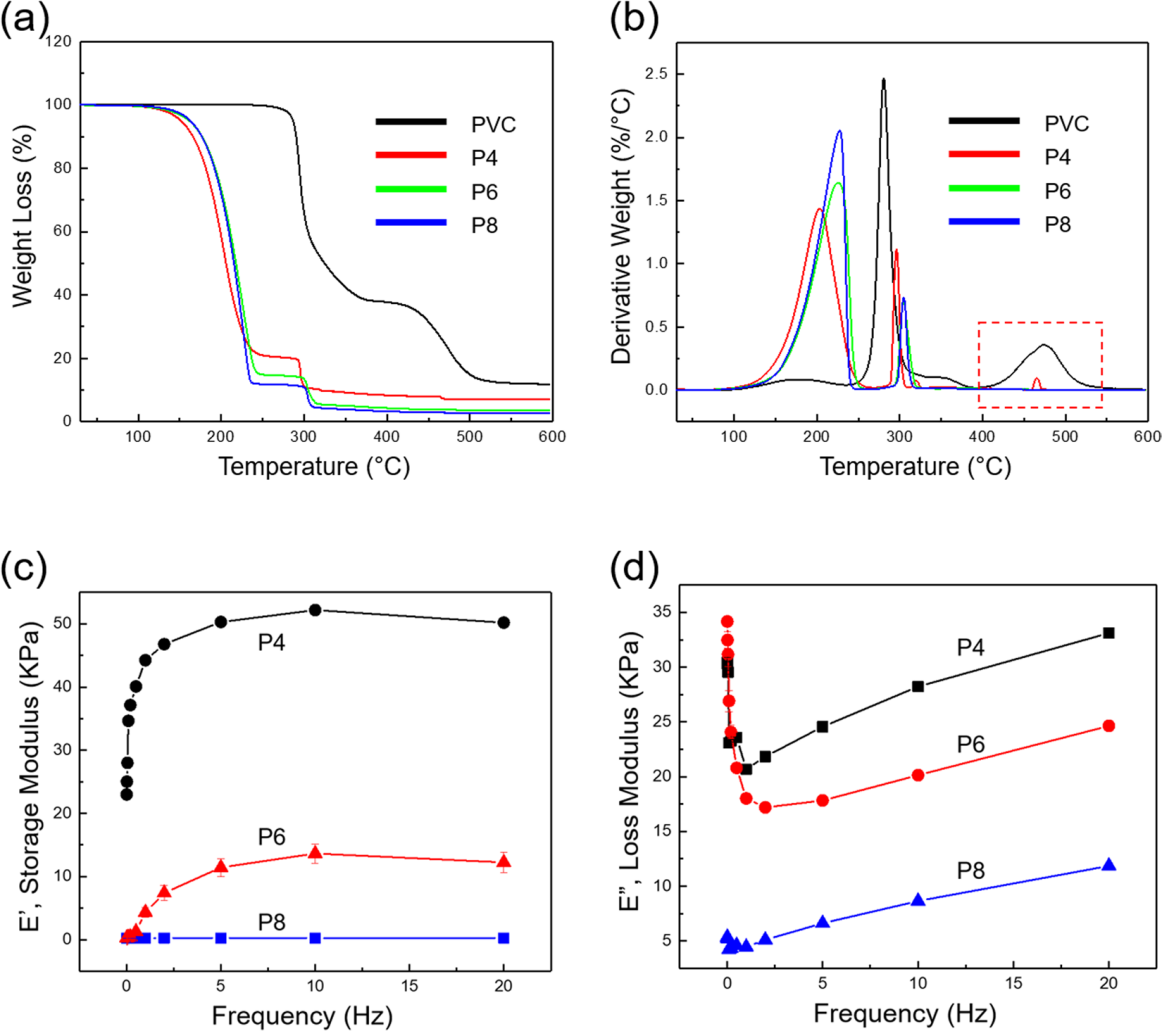
The characterization of PVC and PVC gels for varying PVC/DBA ratios (i.e. 1:4, 1:6 and 1:8): (**a**) TGA and (**b**) TGA derivative curves. (**c**) Storage and (**d**) loss modulus DMA results at room temperature (frequencies ranging 0.01 to 20 Hz).

Figure 2a,b show the thermogravimetric analysis (TGA) and TGA derivative curves for pristine PVC and PVC gels. PVC shows a distinguishable thermal degradation around 235.76–392.30 °C and a weight loss of 62.09%. In the next stage a weight loss of 25.56% is observed, which takes place over the temperature range of 403.31–556.24 °C. PVC gels show major weight loss stages around 104.33–260.77 °C (P4), 94.06–257.04 °C (P6), and 97.19–248.59 °C (P8), respectively. A weight loss of 79.80, 85.50, and 87.96% are observed for P4, P6, and P8, respectively. It is believed that weight loss occurs at this stage due to decomposition of DBA and elimination of organic solvents (THF)^28^. Then, a weight loss of 10.55, 90.6, and 7.19% are shown around 278.94–330.64 °C (P4), 283.28–328.30 °C (P6), and 287.11–321.63 °C (P8), respectively. This seems to result mainly from the decomposition of PVC in the PVC gel. In the TGA derivative curve of P4, there is a small third decomposition around 457.17–476.85 °C (red dot box) due to higher PVC content than the other prepared gels (P6 and P8). The total weight loss is 87.65% (PVC), 91.09% (P4), 94.56% (P6), and 95.15% (P8), respectively. The mechanical properties of PVC gels with different PVC/DBA ratios is studied using Dynamic mechanical analysis (DMA). The samples are cut into circular shapes with a diameter of 10 mm. DMA is tested in compression mode, and samples are oscillated using varying frequencies from 0.01 to 20 Hz at room temperature. Figure 2c,d show the storage and loss modulus values, respectively. In Fig. 2c at 10 Hz, the storage modulus (E′) decreases from 52.18 KPa (P4) to 13.63 KPa (P6) to 0.26 KPa (P8) as the DBA content is increased in the gels. Figure 2d shows the change in loss modulus (E″), where smaller changes are observed than for the storage modulus at 10 Hz: 28.23 KPa (P4), 20.14 KPa (P6), and 8.65 KPa (P8). This demonstrates that the damping properties, defined by (tan δ = *E*″*/E*′)^29,30^, increase with the increasing content of DBA, from 0.54 at a 1:4 PVC/DBA ratio (P4) to 1.48 at 1:6 (P6) to 33.01 at 1:8 (P8). The addition of DBA weakens the interactions between polymer chains, resulting in increased softness and flexibility of gels^31^. These results suggest that both the elastic properties (storage modulus) and the viscous properties (loss modulus) are decreased with addition of DBA in the PVC matrix, which consequently increases the damping properties exhibited by the PVC gels.

**Figure 3:**
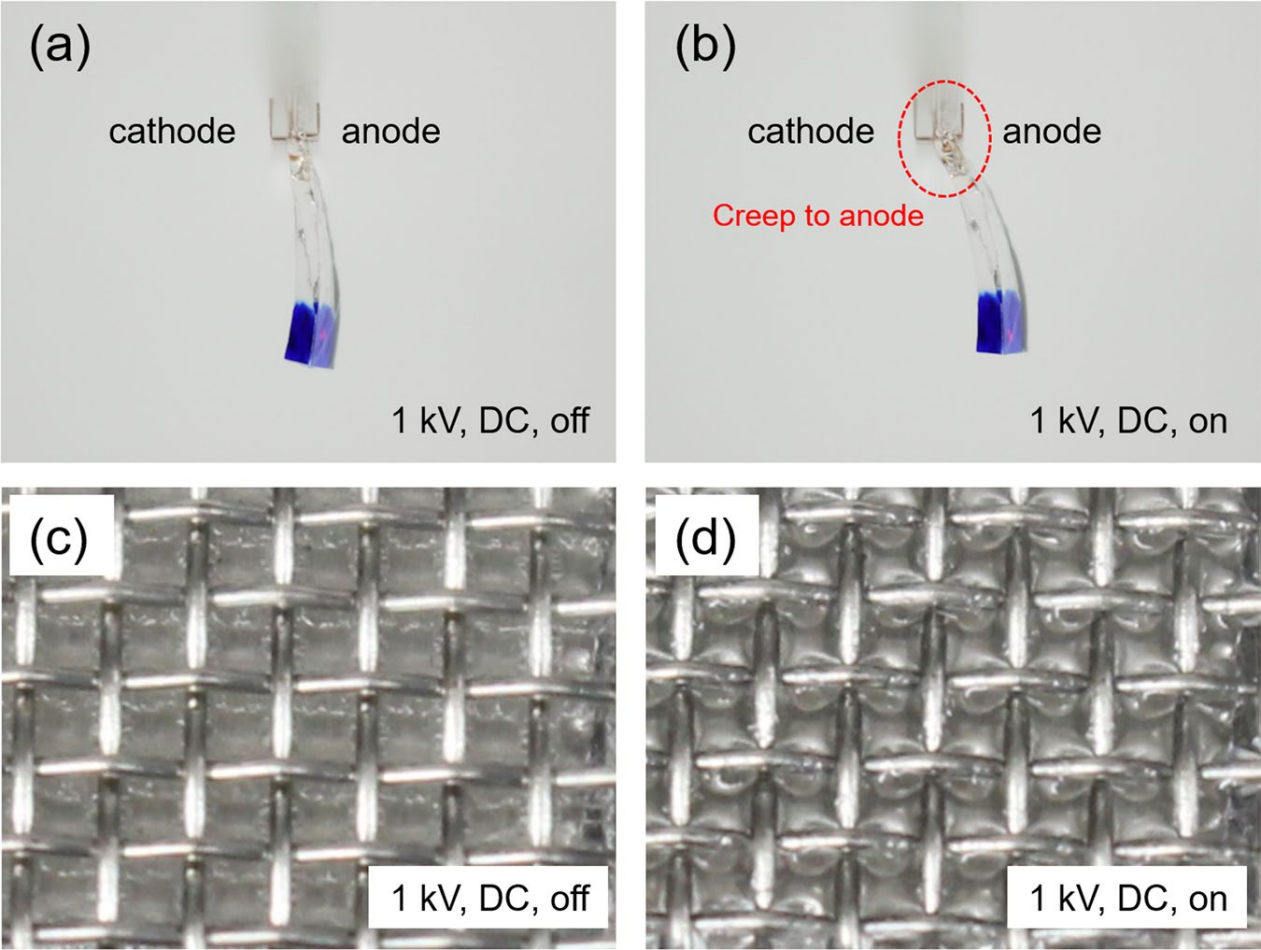
The displacement images of PVC gel: (**a**,**b**) The bending motion of PVC gel with an applied voltage (1 kV, DC) off and on. (**c**) Image of PVC gel without an electric field. (**d**) The PVC gel demonstrates creeping deformation into the vacancies in the anode mesh causing a decrease in thickness and thus actuation with applying an electric field (1 kV, DC).

Figure 3a shows that test setup of the PVC gel actuator, the PVC gel (the bottom of gel is dyed to clearly seen) is bordered by electrodes to demonstrate its bending motion. When a 1 kV electric field with direct current (DC) is applied, electric charges are injected into the gel at the cathode and migrate towards the anode as shown in Fig. 3b. With the removal of the electric field, the PVC gels elasticity causes it to be returned to its initial shape^32^. The charge accumulation on the anode causes the gel to exhibit electrostatic adhesion on the anode surface, and creeping deformation occurs. The bending deformation exhibited by the gel shows not traditional bending motion, but rather bending caused by creeping deformation of the gel onto the anode (Fig. 3b). Figure 3c,d show a contraction type of PVC gel actuator under an applied electric field (1 kV, DC). The PVC gel is placed between a stainless-steel mesh (anode), which is placed on top of the gel, and a stainless foil (cathode) is placed below the gel. The gel shrinks in thickness by creeping up the anode and moving into the empty space of mesh with applying an electric field as shown in Fig. 3d. When the DC field is removed, the gel reverts to its previous shape due to its elasticity. It is also demonstrated that the PVC gel moves in the opposite direction under an alternating current (AC) due to change in charge flow. This can be seen in Video S1 (Supplementary Information).

**Figure 4:**
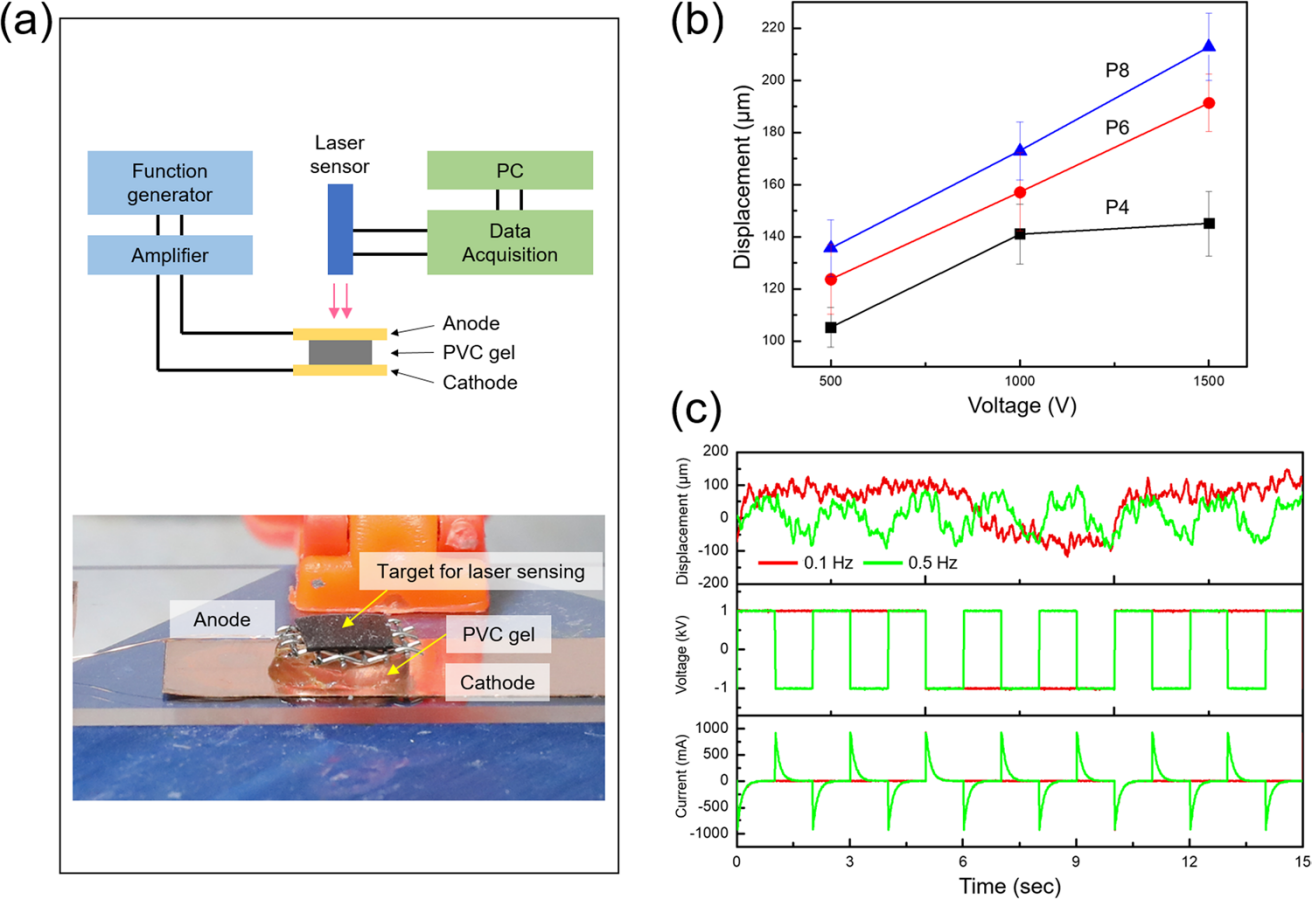
(**a**) Scheme of test set up to measure displacement of single layer PVC gel and image of single layer PVC gel between electrode. (**b**) The displacement results of PVC gel with varied DBA content under electric field (0.5, 1.0, and 1.5 kV with DC). (**c**) The measured voltage, current, and the corresponding displacement responses for the PVC gel using a ± 1 kV, AC, square-wave input with 0.1 and 0.5 Hz frequencies.

Figure 4a shows the scheme of the measurement set up to investigate the displacement for single layer PVC gel actuators with a laser displacement sensor as well as imaging of the single layer actuator. Copper (Cu) tape is used as a cathode and stainless-steel mesh as an anode. Figure 4b shows that the maximum displacement result for PVC gels with varying applied voltage (0.5, 1.0, and 1.5 kV with DC). In Fig. 4b, when the voltage is increased, the maximum displacement for the PVC gel actuators (P4, P6, and P8) is increased from 105.19, 123.67, and 135.55 µm (at 0.5 kV) to 140.93, 157.13, and 172.94 µm (at 1.0 kV) to 145.03, 191.34, and 212.84 µm (at 1.5 kV), respectively. When voltage is increased from 0.5 to 1.0 kV, the displacement of the actuators is increased 34% (P4), 27% (P6), and 28% (P8), which shows that there was not a large amount of variation in relative displacement with increasing plasticizer content. Meanwhile, at the voltage increase interval from 1.0 to 1.5 kV, displacement is increased 3% (P4), 22% (P6), and 23% (P8). These results might suggest that P4 does not show significant change of displacement after 1.0 kV due to its low damping property. Figure 4c shows the responses (voltage, current, and displacement) for sample P8 (PVC/DBA = 1:8) under ± 1 kV, AC (square-wave input with frequencies 0.1 and 0.5 Hz). The displacement is slightly lower at 0.5 than 0.1 Hz. This result is thought to be caused by the insufficient time for polarization to complete gel actuation and restoration time to return to the original shape at higher frequency^33^.

**Figure 5:**
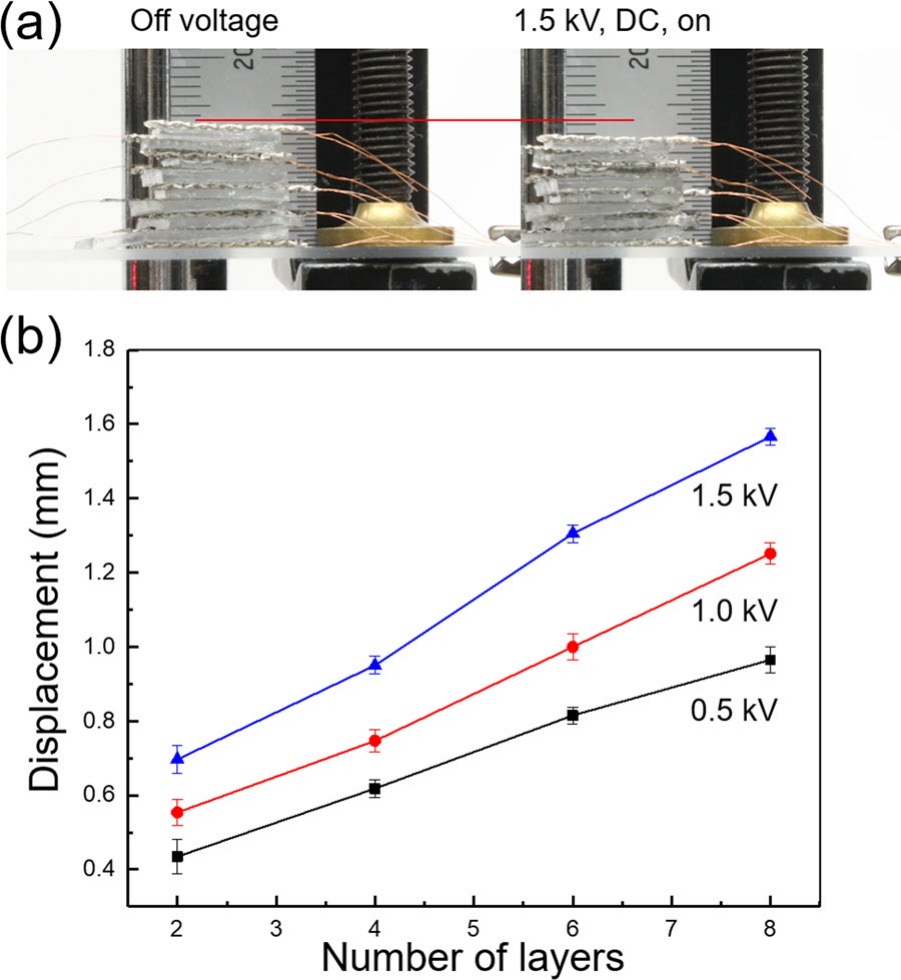
(**a**) Images of 8-stacked PVC gel actuator with/without electric field (1.5 kV, DC). (**b**) The displacement results of stacked PVC gel actuator varied voltage and number of stacked layer.

The stacked PVC gel actuator is prepared to investigate variation of maximum displacement with stacked layers, up to a maximum of 8 layers. The stainless-steel mesh electrodes are used for anodes, while stainless foil (50 µm thick) are utilized as cathodes. The PVC gels (P8) are prepared with dimensions of 2.0 × 1.5 × 0.1 cm^3^ (length × width × thickness). Figure 5a shows the displacement by contracted PVC gels as applying electric field. When electric field (1.5 kV, DC) is applied, the gels shrink in thickness by creeping onto the anode and moving into the empty space of the mesh. A maximum displacement of approximately 1.56 mm is shown. As shown in Fig. 5b, the displacement becomes larger when stacking the PVC gel layers. This result suggests that either more layers, a higher voltage, or both can be used to manipulate the maximum displacement for the stacked PVC gels and the desired displacement can be adjusted with the number of layer or voltage.

**Figure 6:**
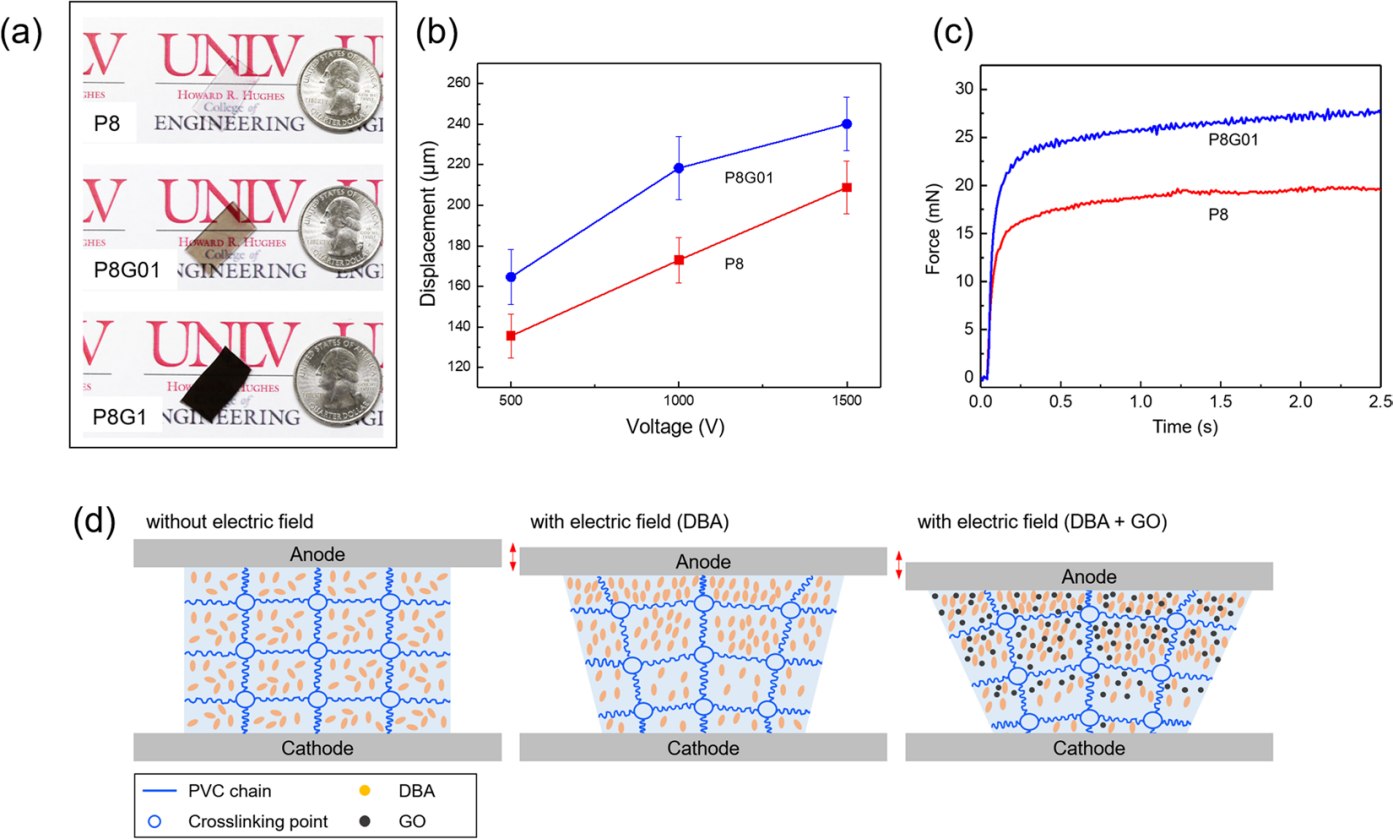
(**a**) Images of prepared PVC and PVC/GO gels. (**b**) The displacement results of PVC and PVC/GO gels under electric field (0.5, 1.0, and 1.5 kV with DC). (**c**) The compression force of PVC and PVC/GO gel under electric field (1.5 kV with DC). (**d**) Scheme of PVC gel internal structure change with an applied electric field.

PVC gels with GO (0.1 wt.% and 1 wt.% of GO to PVC) are prepared to test for possible improvement in electromechanical performance over unaltered PVC gels. Figure 6a shows images of prepared PVC and PVC/GO gels with dimensions of 2.0 × 1.5 × 0.1 cm^3^ (length × width × thickness). However, the 1 wt.% content gel is structurally brittle, due to having too much GO in the PVC gel matrix (a lack of binder), and thus is unable to be utilized for actuation. Figure 6b shows the displacement results of PVC and PVC/GO gel (0.1 wt.%). At 500 V the pristine PVC gel actuator shows a displacement of 135.55 µm, while the PVC/GO shows an enhanced displacement of 164.61 µm (21%). At 1 kV the gels show displacements of 172.94 µm and 218.35 µm (26%). While at 1.5 kV they show displacements of 212.84 µm and 240.13 µm (13%), respectively. This shows an approximate 20% enhancement on average across all voltage ranges for PVC/GO gels over PVC gels. In addition, prepared PVC/GO gel demonstrates faster response than PVC gel under 1 kV, DC, this can be seen in Video S2 (Supplementary Information). The compression force of PVC and PVC/GO gel is investigated under electric field (1.5 kV with DC) as can be seen in Fig. 6c. The PVC/GO gel shows a high level of improvement (27.65 mN) over the plasticized PVC gel actuator (19.66 mN), resulting in a 41% increase in output blocking force. GO contains multiple functional groups such as hydroxyl and epoxy groups^21^. These functional groups allow for electrophoretic mobility of the GO^22,23^. Here this mobility allows for greater creeping movement of the gel due to the movement of GO within the PVC polymer matrix. The effect of DBA and GO movement in PVC gel can be seen in Fig. 6d. The polarized DBA and GO orient themselves in the direction of the electric field. When applying a field to the PVC gel, an electrostatic force acts on the internal structure of gel which results in pulling of the cross-linking points in the PVC molecules by orientation and movement of DBA and GO^17^.

**Figure 7:**
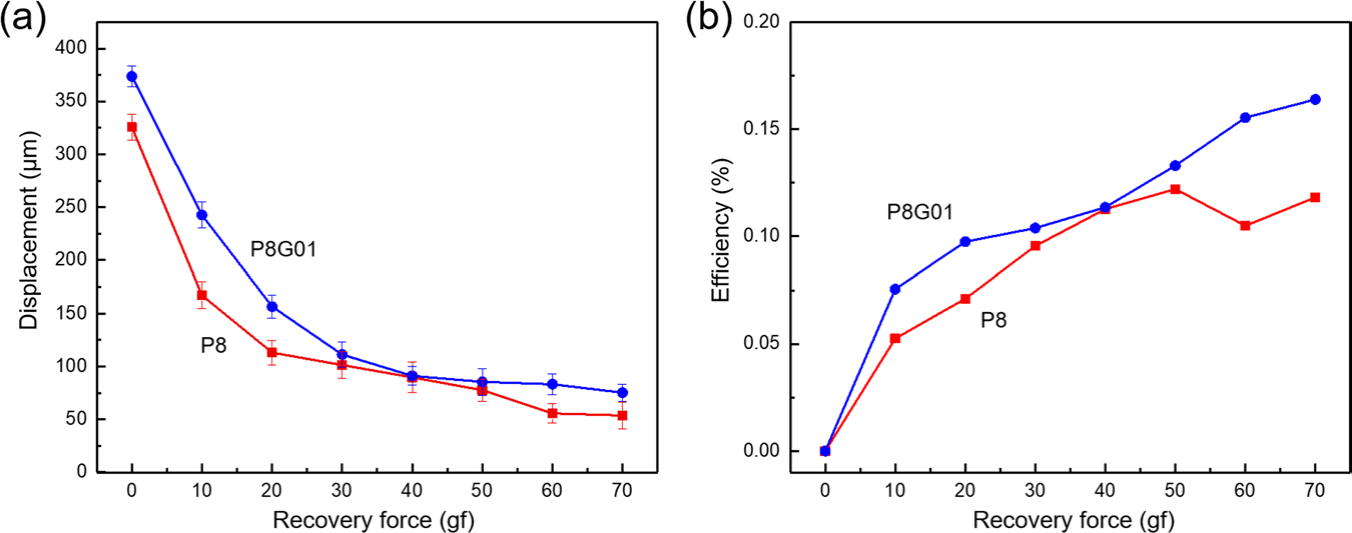
(**a**) The displacement and (**b**) efficiency results of prepared PVC and PVC/GO gels with increasing weight on the top of single layer of gel actuator (applied 1.5 kV with DC).

While the contraction force was measured and presented, the recovery force is more easily utilized for maximum force output^34^. A single layer of both PVC and PVC/GO gels are tested. The force output is measured along with the displacement variation by applying weight on the top of PVC and PVC/GO gels (using test masses from 0 to 70 g), as shown in Fig. 7a. The PVC/GO gel has a marginally larger output displacement for all output forces. The measured and estimated results are summarized in Table 2. The maximum recovery force for both actuators is 686 mN at 1.5 kV. The current for PVC and PVC/GO gels is also measured and found to be approximately 0.104 and 0.105 mA at 1.5 kV, respectively. The maximum power output for the PVC and PVC/GO gels is found to be 0.190 and 0.258 mW, respectively. The PVC/GO gel actuator demonstrates an increase of power output of 35.6% over the PVC gel actuator. The estimated efficiency of actuators is shown in Fig. 7b. Both actuators have been optimized for maximum displacement, and this results in suboptimal energy efficiency. The current is found to vary non-linearly with voltage (the current when actuated with 400 V was only 0.012 mA; 11.3% of the current at 1.5 kV), the force output would be increased with a larger surface area for the actuator, and the overall displacement of PVC gel actuators has been shown to scale linearly with the number of stacked layers whereas the current does not^34^. These are all areas which can be easily altered to achieve a higher force and higher efficiency actuator if so desired.

**Table 2:** The summarized results of power and efficiency of PVC and PVC/GO gels.

Sample	Weight (g)	Force (mN)	Current (mA)	Power input (mW)	Displacement(µm)	Power output (mW)	Efficiency (%)
P8	−	−	0.104	156.0	325.677	−	−
	10	98			167.161	0.082	0.053
	20	196			113.090	0.111	0.071
	30	294			101.290	0.149	0.096
	40	392			89.650	0.176	0.113
	50	490			77.640	0.190	0.122
	60	588			55.650	0.164	0.105
	70	686			53.710	0.184	0.118
P8G01	−	−	0.105	157.5	373.580	−	−
	10	98			242.580	0.119	0.076
	20	196			156.580	0.154	0.097
	30	294			111.194	0.164	0.104
	40	392			91.160	0.179	0.114
	50	490			85.410	0.209	0.133
	60	588			83.190	0.245	0.155
	70	686			75.190	0.258	0.164

**Figure 8:**
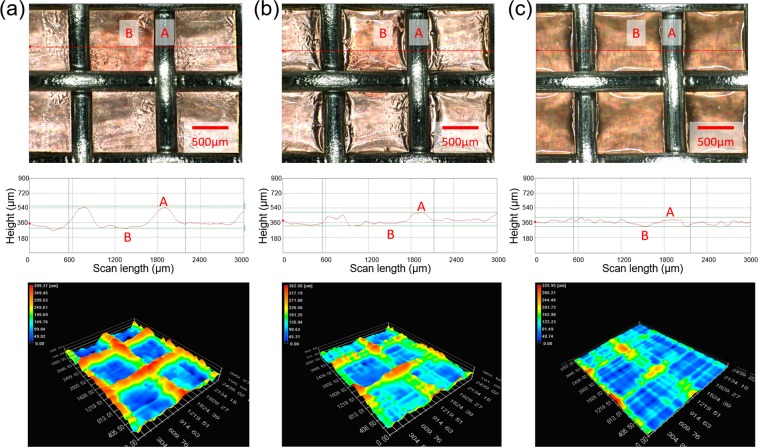
Images of top view, height profile, and topographic views of prepared PVC and PVC/GO gels. (**a**) P8 without an applied electric field. (**b**) P8 with an applied electric field, (**c**) P8G01 with an applied electric field (1.5 kV with DC).

Figure 8 shows that images of top view (top), height profile (middle), and topographic views (bottom) of prepared PVC and PVC/GO gels. Prepared gels were examined using 3D microscope to visualize displacement of PVC and PVC/GO gel by comparing the height between the top of stainless-steel mesh (A) and top of gel (B) before and after applying an electric field. The height of P8 before, P8 after, P8G01 after applying an electric field (1.5 kV with DC) was measured 273.9, 170.1, and 115.7 µm, respectively. As a result, the topographic view of Fig. 8a shows clear orange-colored mesh pattern due to the large height difference. When an electric field is applied to specimens, the height difference is reduced because of gel creep to the anode (stainless-steel mesh) as can be seen in Fig. 8b,c. It would be expected that the addition of GO in PVC gel would show a larger creeping movement than DBA only. In future work, further study for GO in PVC gels can be conducted on PVC/GO gel characterization as well as research of the promising potential improvement of artificial muscles.

## Conclusions

In this paper, the thermal and mechanical properties, the chemical structure of PVC gels with varied PVC to DBA ratios (i.e., 1:4, 1:6 and 1:8), and the effects of GO inclusion on gel deformation were presented. The PVC gel actuators (single and multi-stacked) electromechanical performance was demonstrated under electric fields from 0.5 to 1.5 kV. When the content of DBA in PVC gel was increased, the gel showed higher electromechanical performance (increases displacement). The inclusion of GO (0.1 wt.%) was shown approximate 20% enhancement of displacement performance on average across all voltage ranges for PVC/GO gels over existing plasticized PVC gels and the 41% increase in blocking force and 36% increase in power output are highly significant. These results show that GO addition in the PVC gel matrix could prove highly valuable in applications such as optics, small mechanical devices, artificial muscles, and various opto-electro-mechanical devices. In addition, we believe that additional steps can enhance the performance of PVC gel. Further work will be focused on the investigation of different shapes, modified materials, or electrode configurations to improve their performance for various applications.

## Methods

### Materials and PVC gel and PVC/GO gel preparation

The gels were prepared using commercially purchased PVC (Mw = 80,000 g mol^−1^, Mn = 47,000 g mol^−1^), DBA (M_W_ = 258.38 g mol^−1^), and THF, which were purchased from Sigma-Aldrich Co. The ratios of PVC to DBA were composed to 1:4, 1:6, and 1:8. The PVC was dissolved in THF and DBA solution with stirring at 50 °C for 4 hr. And then, the solution was casted in a glass Petri dish. THF was removed by evaporation in the fume hood (at room temperature for 4 days). PVC/GO gels were prepared using functionalized GO, which was purchased from Graphene Supermarket, US. GO was dispersed into THF by ultra-sonication (VC-505, Sonics) for 3 hr., then PVC/GO gels were prepared following above mentioned process to prepare PVC gels (with a 1:8 PVC/DBA ratio). The thickness of both PVC gels and PVC/GO gels were around 1 mm, approximately. The conditions to prepare gels are summarized in Table 1. Fig. S1a shows the chemical structure of PVC and DBA and Fig. S1b shows image of prepared gel which is transparent and flexible. The images of prepared PVC and PVC/GO gels can be seen in Fig. S1c (Supplementary Information).

### Preparation of PVC and PVC/GO gel actuator

Stainless steel mesh was used as anode for contraction type PVC gel actuator. The gel is placed between a stainless steel mesh (anode) and a stainless steel foil or Cu tape (cathode).

### Characterizations

The chemical structure of the specimens was studied using a Fourier transform infrared spectrometer (FT-IR; IRTracer-100, Shimadzu) and an attenuated total reflectance accessory (MIRacleTM, PIKE Technologies). TGA (Q500, TA instruments) was conducted to investigate the thermal properties of the gels with a nitrogen atmosphere and heating rate of 10 °C min^−1^ up to 600 °C. DMA was tested to characterize the mechanical properties with Pyris Diamond DMA (Perkin-Elmer) and analysis program (Seiko Instruments Inc.) at room temperature. The DMA frequencies were adjusted from 0.01 to 20 Hz. 3D measurement of specimens was carried out using VHX-100 (Keyence) with x50 magnification. The electromechanical responses (displacement and blocking force) were measured using a laser displacement sensor (optoNCDT-1401, Micro-Epsilon) and a load cell (GSO-100, Transducer Techniques) with a test setup using a signal generator (SDG-1025, Siglent), a high voltage amplifier (609E-6, Trek), and data acquisition system (SCB-68, National Instruments). The responses (voltage, current, and displacement) were analyzed using a different DAQ (LabView 8, National Instruments and DAQ-6510, Keithley).

## Supplementary information

Supplementary Information

S1 PVC gel (P8) movement

S2 PVC and PVCGO gels movement
