# Supplementary material for: High-performance polyvinyl chloride gel artificial muscle actuator with graphene oxide and plasticizer
Source: Sci Rep. 2019 Jul 4;9:9658. doi: 10.1038/s41598-019-46147-2 (PMC6609716; doi:10.1038/s41598-019-46147-2)
Supplement: Supplementary file 1 — Supplementary Information [file 41598_2019_46147_MOESM1_ESM.docx]

Supplementary Information

High-performance polyvinyl chloride gel artificial muscle actuator with graphene oxide and plasticizer

Taeseon Hwang, Zachary Frank, Justin Neubauer, and Kwang Jin Kim ^[[1]](#footnote-1)^*

*Department of Mechanical Engineering, University of Nevada, Las Vegas, 4505 Maryland Parkway, Las Vegas, Nevada 89154, United States*


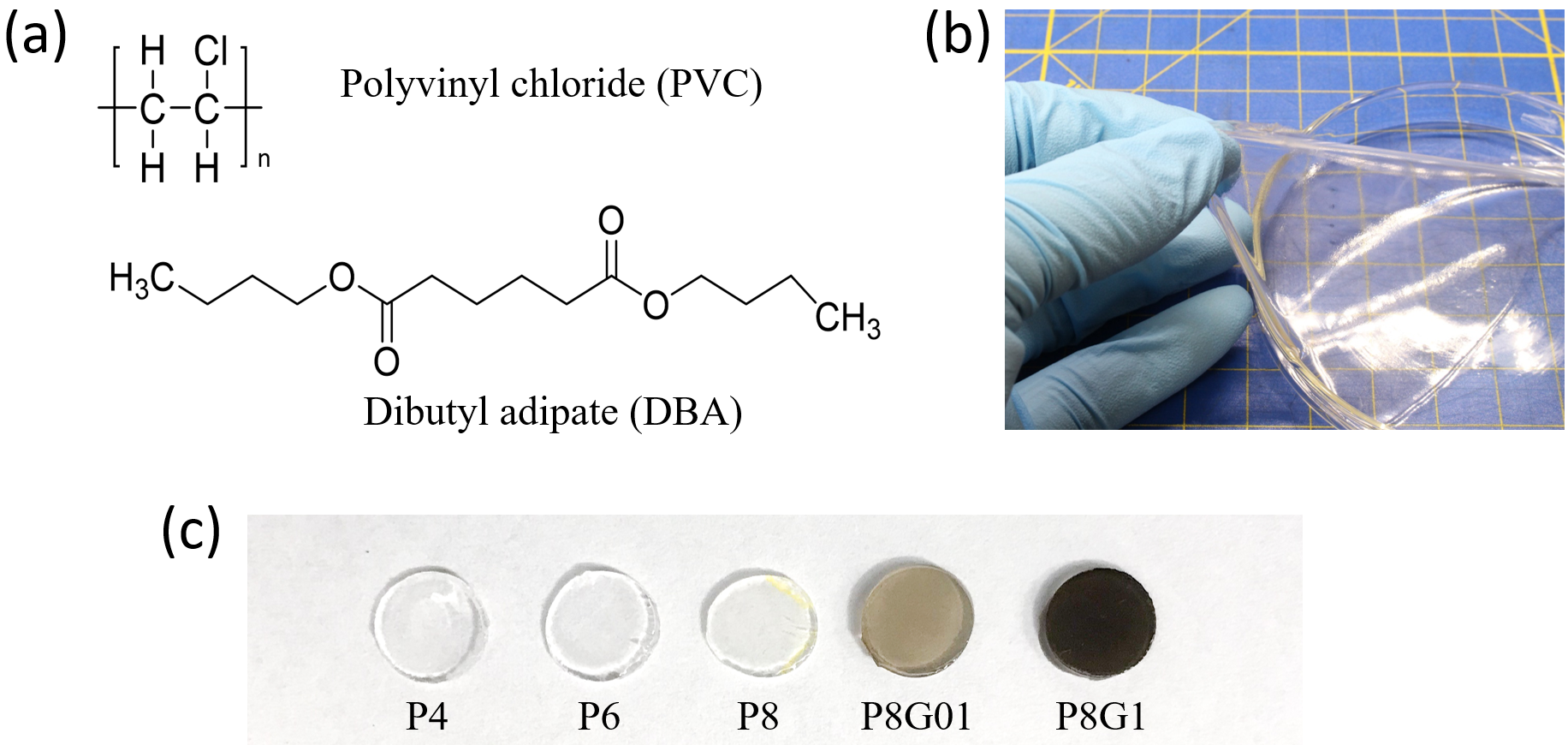


**Figure S1.** a) The chemical structure of PVC and DBA. b) Photograph of a prepared PVC gel (P8) by casting method, shows transparent and has good flexibility. c) Image of prepared PVC and PVC/GO gels (sample names are shown in the below).

**Video S1.** PVC gel (P8) movement in opposite direction under applying ±1 kV, AC.

**Video S1.** PVC and PVC/GO gels displacement under same electric field (1 kV, DC).

1. * Corresponding author at: Active Materials and Smart Living Laboratory, Department of Mechanical Engineering, University of Nevada-Las Vegas, 4505 Maryland Parkway Las Vegas, NV 89154-4027, USA. Phone: +1 702 774 1419. E-mail address: kwang.kim@unlv.edu (K. Kim). (www.kwangjinkim.org) [↑](#footnote-ref-1)
